# The Structure of Human Prions: From Biology to Structural Models — Considerations and Pitfalls

**DOI:** 10.3390/v6103875

**Published:** 2014-10-20

**Authors:** Claudia Y. Acevedo-Morantes, Holger Wille

**Affiliations:** Department of Biochemistry and Centre for Prions and Protein Folding Diseases, University of Alberta, Edmonton, AB T6G 2M8, Canada; E-Mail: cacevedo@ualberta.ca

**Keywords:** prion, cellular prion protein, PrP^C^, misfolded prion protein, PrP^Sc^, *PRNP*, amyloid, α-helices, β-sheets

## Abstract

Prion diseases are a family of transmissible, progressive, and uniformly fatal neurodegenerative disorders that affect humans and animals. Although cross-species transmissions of prions are usually limited by an apparent “species barrier”, the spread of a prion disease to humans by ingestion of contaminated food, or via other routes of exposure, indicates that animal prions can pose a significant public health risk. The infectious agent responsible for the transmission of prion diseases is a misfolded conformer of the prion protein, PrP^Sc^, a pathogenic isoform of the host-encoded, cellular prion protein, PrP^C^. The detailed mechanisms of prion conversion and replication, as well as the high-resolution structure of PrP^Sc^, are unknown. This review will discuss the general background related to prion biology and assess the structural models proposed to date, while highlighting the experimental challenges of elucidating the structure of PrP^Sc^.

## 1. Introduction

The prion protein can exist in multiple isoforms, predominantly the cellular, non-infectious PrP^C^ and the disease-causing PrP^Sc^. The prion protein is most highly expressed in the central nervous system (CNS), but it can be found in other tissues and cell types as well [1,2,3].

The misfolded PrP^Sc^ causes the prion diseases, also known as transmissible spongiform encephalopathies (TSEs), a group of progressive, neurodegenerative disorders in humans that include kuru [4], Creutzfeldt-Jakob disease (CJD) [5], Gerstmann-Sträussler-Scheinker (GSS) syndrome [6], and fatal familial insomnia (FFI) [7,8]. PrP^Sc^ is also responsible for chronic wasting disease (CWD) in cervids [9,10]; bovine spongiform encephalopathy (BSE) in cattle [11,12,13,14], and its analogues in antelopes and wild felids [15,16,17]; scrapie in sheep, mufflons [18], and goats [19]; transmissible mink encephalopathy (TME) in ranch-reared mink [20]; and feline spongiform encephalopathy (FSE) in domestic cats [21,22].

The key molecular event in the pathogenesis of the prion diseases is the conformational conversion of PrP^C^ into PrP^Sc^. In a process that is not fully understood, PrP^Sc^ binds to PrP^C^ and promotes its transformation into PrP^Sc^. Eventually, the abnormal protein isoform leads to neurodegeneration and cell death, and as a consequence many microscopic, sponge-like holes (vacuoles) can be seen in the brain, a symptom of prion disease.

Scrapie was the first prion disease to be identified in the 1730s. Later other prion diseases were described: GSS (1920s); CJD (1920s); kuru (1952–1953, among the Fore people of Papua New Guinea, transmitted via ritualistic cannibalism); CWD (1967); and the most recent major animal disease: BSE (1987). Prion diseases are classified as: (1) sporadic (arise spontaneously for no known reason, with an incidence 1 per 10^6^ population per year); (2) inherited (with an incidence of 1 per 10^7^–10^8^ population per year); and (3) acquired (by medical procedures or contaminated food). Prion diseases occur worldwide and affect both genders equally.

## 2. The Human Prion Protein Gene (*PRNP*)

The human *PRNP* gene is located on the short arm of chromosome 20 between the end of this arm and the position 12 (p12-pter). The structure of the prion gene for all species of mammals studied to date contains three exons. The open reading frame (ORF) lies entirely within exon 3 and transcribes an mRNA of 2.1–2.5 kb in length [23,24,25], with approximately 50 copies/cell in neurons [24]. To date, there are no reported prion disease-associated mutations in either exon 1 or 2, or within any of the introns.

Codons 51 to 91 of the *PRNP* gene encode a nonapeptide (PQGGGGWGQ) followed by four octapeptide repeats (PHGGGWGQ), which are almost identical to the nonapeptide except for the omission of a glycine and the presence of a histidine instead of a glutamine at the second position. A variety of insertion mutations have been found in the octapeptide repeat region. These insertions so far comprise one to nine extra octapeptide repeats [26]. Raman spectroscopy studies have demonstrated that the binding of copper ions (Cu^2+^) to the octapeptide repeats in PrP is coordinated by histidine residues, suggesting that this region may be involved in the transport of extracellular Cu^2+^ ions to an endosomal compartment [27].

Mutations in the *PRNP* gene can cause the development of prion diseases and lead to different clinical phenotypes, including CJD, GSS and FFI [28,29,30]. Mutations of the prion protein gene can be classified as: (1) point mutations (*i.e.*, single nucleotide substitutions), which can cause an amino change (missense mutation), can be silent (do not cause alteration in the amino acid sequence), or less commonly can cause the coding to prematurely terminate (stop or nonsense mutation); and (2) insertions and deletions, which are associated with prion diseases.

## 3. Evolutionary Origins of *PRNP*

Recently it has been proposed that the emergence of the prion protein founder gene was based on two genomic rearrangements that occurred hundreds of millions of years ago [31]. The first event occurred within the *N*-terminal ectodomain of a ZIP predecessor gene, when metazoans first emerged on the planet. As a consequence of this rearrangement a cysteine-flanked core (CFC) region was generated within this ectodomain. The second event, which allowed the creation of the prion protein founder gene to be traced back, occurred approximately a half-billion years ago (around the Cambrian explosion). This later event resulted in the apparent genomic retro-insertion of a spliced and *C*-terminally truncated ZIP gene transcript, indicating that the prion protein founder gene is equivalent to a retrogene [32].

An earlier investigation using a quantitative interactome analysis identified ZIP6 and ZIP10 as potential molecular interactors of PrP at the cell surface, revealing that the known prion proteins are structurally related to an extracellular domain of ZIP6 and ZIP10 [33]. Similarly, other authors using interactome analyses have identified multiple interactions between PrP^C^ and other molecules, like the neural cell adhesion molecule (NCAM) [34], the laminin receptor precursor [35], Na/K ATPases [36] and protein disulfide isomerases (PDI) [37]. Together, these findings suggest that PrP^C^ organizes its molecular environment by binding adhesion molecules, which in turn recognize oligomannose-bearing membrane proteins.

## 4. Polymorphisms and Mutations in the *PRNP* Gene ORF

**Figure 1:**
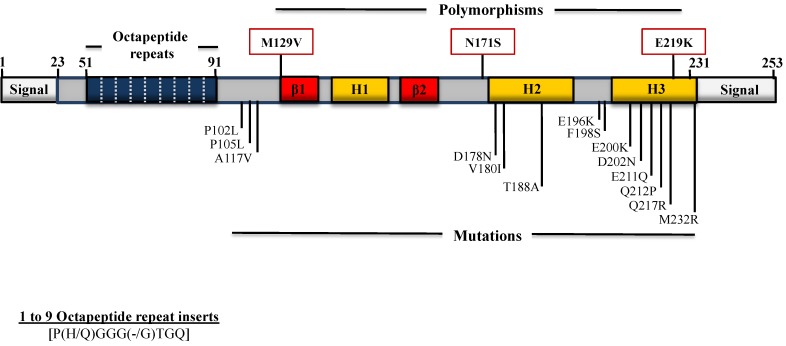
Schematic representation of human PrP mutations and polymorphisms. The 0.76 kb ORF of the *PRNP* gene encodes a 253 amino acid protein, PrP^C^. Human PrP consist of a cleaved signal peptide (1–22), an octapeptide repeat-containing unfolded domain (OR, 51–91), three α-helices (H1, H2, and H3), one small, antiparallel β-sheet (β1 and β2), and a GPI-anchor signal (232–253). The *N*-terminal octapeptide repeat motif is comprised of eight residues: P(H/Q)GGG(-/G)WGQ. Normal PrP contains five copies of this motif; a single OR deletion is considered a non-pathogenic polymorphism. However, insertional mutations consisting of one to nine additional OR are pathogenic. Polymorphisms and pathogenic mutations of the *PRNP* gene are represented above and below the schematic, respectively. Letters preceding the numbers indicate the normal amino acid residue for the position and letters following the numbers designate the new residue.

More than 30 mutations in the *PRNP* gene have been linked to inherited prion diseases, including CJD, GSS, and FFI [38]. In addition, several common polymorphisms (variations) were identified in the *PRNP* gene as well (Figure 1). Mutations can lead to a change of a single amino acid in PrP, insert additional residues, or cause an abnormally short version of PrP to be expressed. These mutations affect the primary structure of PrP, may lead to changes in the secondary and tertiary structure, and could result in the emergence of PrP^Sc^ confomers. In contrast, polymorphisms do not cause prion disease, but may influence a person’s risk of developing a prion disease.

The most common and best-studied polymorphism in human PrP occurs at codon 129 and acts as a predisposing factor for sporadic, iatrogenic, and variant CJD [39,40]. Either methionine (M) or valine (V) is encoded at this position. M/M homozygosity at this position appears to be associated with an earlier age of onset and rapidly progressive dementia; whereas a more prolonged course with an ataxic onset is most often associated with the V/V allele. It is important to highlight that the allele frequencies at codon 129 differ across populations, for example compared with Caucasian frequencies of M(0.66):V(0.34), the allelic frequencies reported in Japan are M(0.96):V(0.04), and although the incidence of disease has not been reported to be higher in Japan, it may predispose an individual to develop a particular clinical phenotype [41].

While M or V homozygosity at codon 129 in human PrP results in a predisposition to sporadic or iatrogenic CJD, the glutamate (E) to lysine (K) substitution at codon 219 appears to have a protective effect against sCJD. The E219K polymorphism has been reported to occur in the Japanese population with an allelic frequency of 6% [42]. This polymorphism was also reported on the same allele as the P102L mutation (see below) in a Japanese family in which dementia rather than ataxia was prominent and cerebellar plaque pathology was less prominent compared with cases of the P102L mutation alone [43]. Recent studies in knock-in mice have shown that a heterozygous state at codon 219 confers reduced susceptibility to prion infection [44].

### 4.1. Mutations Associated with GSS

This subtype of prion disease is very rare and associated with autosomal-dominant inheritance. The clinical features include ataxia of gait and/or dysarthria, dementia, spastic paraparesis, and variable degrees of pyramidal and extrapyramidal symptoms [45]. The incidence is not clear but was estimated to be 1 to 10 per 100 million. GSS has been associated with the following missense mutations: P102L [46]; P105L [47]; A117V [48]; F198S [49]; D202N, Q212P [50]; Q217R [49], and in some cases of octapeptide repeat insertions (OPRI), especially those with larger inserts (approximately 192 bp) [51,52,53].

### 4.2. Mutations Associated with CJD

CJD is another neurodegenerative disorder characterized by failing memory, behavioural changes, lack of coordination, and visual disturbances. As the disease progresses, mental deterioration becomes more pronounced, jerky movements, blindness, and coma may occur. This disease leads to death usually within 1 year of onset of illness. There are three major categories of CJD:

- ***Sporadic CJD (sCJD)*:** the most common type of CJD, accounting for at least 85% of cases. The disease manifests even though the person has no known risk factors for the disease (e.g., no mutation in the *PRNP* gene);
- ***Familial CJD (fCJD)*:** accounting for about 5% to 10% of cases of CJD. The individual has a family history of the disease and/or tests positive for a *PRNP* mutation;
- ***Iatrogenic CJD (iCJD)*:** accounting for less than 1% of CJD cases. This form of CJD is transmitted by exposure to brain or tissue from an infected person, usually through a medical procedure, such as a blood transfusion or dura mater transplant.

fCJD has been associated with the following missense mutations: D178N [54], V180I [47], T188A [55], E196K [56], E200K [57], V203I [56], R208H [58], V210I [59], E211Q [56], M232R [47]; and one to nine octapeptide inserts [51,60,61,62].

### 4.3. Mutations Associated with FFI

Fatal familial insomnia (FFI) is a rare genetic sleep disorder, diagnosed in less than 40 families worldwide. This disease is caused by the missense mutation D178N [8,63], but it can also be developed spontaneously in patients with a sporadic variant called sporadic fatal insomnia (sFI). The clinical profile includes intractable insomnia that may be observed for several months prior to the obvious onset of disease, which may include dysautonomia, ataxia, variable pyramidal and extrapyramidal signs and symptoms, and relatively spared cognitive function until late in the course [40]. The average survival for FFI patients after the onset of symptoms is 18 months [64].

## 5. Biochemistry and Structure of Prion Proteins

Since the discovery of prions, many attempts have been made to elucidate the structural differences between PrP^C^ and PrP^Sc^ and the pathogenic conversion that leads to the formation of PrP^Sc^. Low-resolution biophysical and biochemical techniques have been used by many research groups to gain insights into the conformations of both PrP^C^ and PrP^Sc^, however these approaches have limitations as will be discussed below.

PrP^C^ and PrP^Sc^ differ regarding their solubility, fibril formation tendency, and proteinase K resistance. PrP^C^ is a soluble protein with a high susceptibility to proteolytic digestion, whereas PrP^Sc^ is characterized by its insolubility in detergents and partial resistance to proteolysis in its aggregated form [65,66,67]). These differences appear to be based on their structural differences, since previous studies based on circular dichroism and infrared spectroscopy have shown that PrP^C^ is dominated by α‑helices (42%) and has only a small fraction of β-sheet content (3%) [67], while PrP^Sc^ contains predominantly β-sheets (>43%) [67,68]. The *N*-terminally truncated PrP^Sc^, designated PrP 27–30 has a higher β-sheet content (>54%). In contrast to PrP 27–30, which polymerized into rod-shaped amyloids, PrP^C^ does not form aggregates detectable by electron microscopy [67,69].

### 5.1. Structure of PrP^C^

**Figure 2:**
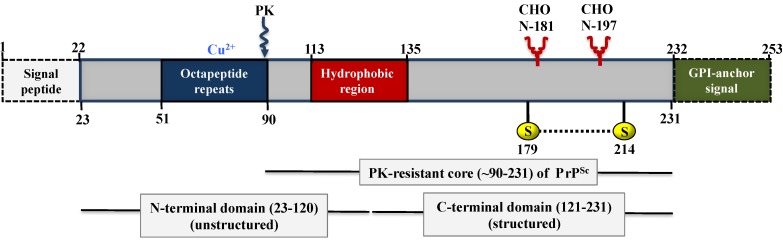
Organization of human PrP. The unprocessed PrP is 253 amino acid residues in length and includes a signal peptide (1–22), four OR, a hydrophobic region (113–135), one disulphide bond between cysteine residues (179–214), two *N*-linked glycosylation sites (at residues 181 and 197), and a GPI-anchor attached to the *C*-terminus of PrP replacing the GPI-anchor signal (residues 232 to 254). The four OR in the *N*-terminal domain have a high affinity for copper ions (Cu^2+^), while a preceding nonapeptide (PQGGGGWGQ) lacks the histidine that is necessary to bind a Cu^2+^ ion. Mutated forms of PrP can contain insertions of one to nine additional OR or a deletion of one OR. A palindromic region, AGAAAAGA (113–120), lies in the hydrophobic region (113–135) and is thought to be important in the conversion of PrP^C^ to PrP^Sc^. OR: Octapeptide repeat; GPI: glycophosphatidylinositol; PK: proteinase K; CHO: carbohydrates.

The prion protein exists in multiple conformations and its cellular isoform, PrP^C^, which is found in healthy organisms, is among the most extensively studied proteins. In humans, the newly synthesized and unprocessed PrP^C^ is approximately 253 amino acids in length and has a molecular weight of 35–36 kDa (Figure 2). PrP^C^ is GPI-anchored and resides in rafts at the cell membrane. After ~5 h at the cell surface (its average half-life) it is internalized through a caveolae-dependent mechanism and is degraded in the endolysosome compartment. It has been speculated that the PrP^C^ conversion to PrP^Sc^ may occur in caveolae-like domains [70,71].

The structure of PrP^C^ could not be studied using NMR spectroscopy or X-ray crystallography due to the low yield of protein that is obtained during the purification process [67,69]. This limitation was overcome by using recombinant PrP (recPrP) as a surrogate for PrP^C^, since NMR studies have shown that recPrP appears to have the same molecular architecture as PrP^C^ [72,73,74]. A first attempt to predict the three-dimensional structure of PrP^C^ was based on circular dichroism and infrared spectroscopy measurements [75], proposing a X-bundle α-helical structure. Later the groups of Wüthrich [72,76] and James [77] determined the structure of the *C*-terminal, folded domain of PrP (amino acids 121–231) by NMR spectroscopy. For all species analyzed, the structure of recPrP consists of three α-helices (amino acids 144–154, 175–193 and 200–219) and a small antiparallel β-sheet (amino acids 128–131 and 161–164) [78]. It has an amino-terminal signal peptide (amino acids 1–23), a more or less flexible *N*-terminal domain (amino acids 23–120), a folded *C*-terminal domain (amino acids 121–231), and a signal peptide for membrane attachment via a glycophosphatidylinositol (GPI) anchor (amino acids 232–254). It contains a single disulfide bond linking the cysteine residues at positions 179 and 214, connecting helices 2 and 3 [69,79] and two *N*-linked glycosylation sites at asparagines 181 and 197.

The *N*-terminal domain contains an eight amino acid repetitive motif comprised of the residues PHGGGWGQ, which has a high affinity for copper ions (Cu^2+^) [80,81,82]. Therefore, it has been hypothesized that PrP^C^ is involved in copper metabolism [83], but its ancestral relationship to the ZIP proteins also suggests functional interactions with zinc ions (Zn^2+^) [33]. The latter assumption is supported by recent findings from Glenn Millhauser’s group suggesting that electrostatic interactions stabilize the PrP-Zn^2+^ complex. These results indicate that Zn^2+^ drives a tertiary structure contact between the Zn^2+^-bound octapeptide and the *C*-terminal domain surface that includes many of the PrP mutations that give rise to familial prion diseases [84]. Other research findings have suggested that the octapeptide repeats and the *N*-terminal polybasic region in PrP^C^ mediated the zinc influx into neuronal cells via α-amino-3-hydroxy-5-methyl-4-isoxazolepropionate (AMPA) receptors, with PrP^C^ acting as a zinc sensor and the AMPA receptor as a zinc transporter. These results also suggest that PrP-mediated zinc uptake may contribute to neurodegeneration in prion and other neurodegenerative diseases [85].

**Figure 3:**
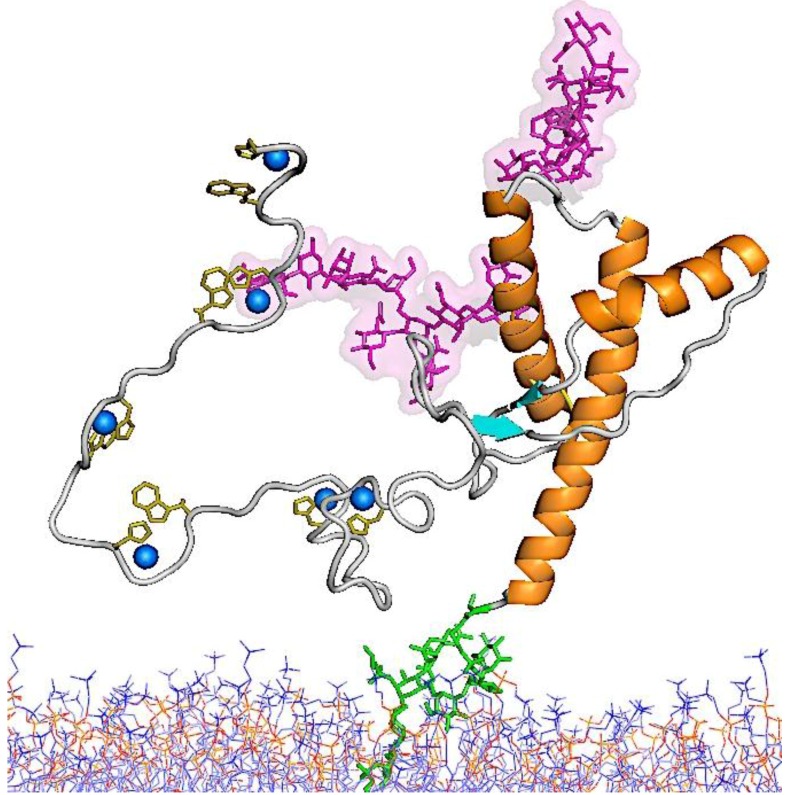
Schematic diagram of the structure of PrP^C^. The carbohydrate moieties that are linked to Asn 181 (down) and Asn 197 (up) are shown in pink. The *C*-terminal GPI-anchor is shown in green and is extending into the cell membrane in blue and red. OR residues in the *N*-terminal domain are known to bind copper ions (shown in blue). The composite data file for the structure [90,91] was kindly provided by Dr. Glenn Millhauser (University of California, Santa Cruz) and includes coordinates for the carbohydrates, the GPI-anchor, and the membrane that were provided by Dr. Valerie Daggett (University of Washington, Seattle).

A hydrophobic sequence in the middle of the protein (amino acids 113–135) may serve as a transmembrane domain in some prion protein isoforms [86]. Within this sequence stretch lies a palindromic region (113–120) consisting of an alanine-rich tract of amino acids, AGAAAAGA, which is highly conserved across a wide variety of mammalian species. Previous studies targeting this region have shown that deletion of this palindrome prevents conversion of both mutant and co-expressed wild-type (wt) PrP [87,88]. Moreover, peptides generated from this palindrome interfere with *in vitro* formation of PK-resistance [89]. Figure 3 shows a schematic representation of PrP^C^-bound to the cell membrane [90,91].

### 5.2. Structure of PrP^Sc^

Despite a large interest in determining the structure of PrP^Sc^, little is known about the molecular details of this isoform [92]. The high-resolution structure of PrP^Sc^ and its proteolytically truncated variant, PrP 27–30, have evaded experimental determination due to their insolubility and propensity to aggregate. However, it is widely accepted that: (1) during the conversion of PrP^C^ to PrP^Sc^ the β-strand content increases substantially [68,93]; (2) fibrillar assemblies of PrP^Sc^ display a typical cross-β sheet architecture, which is characteristic for amyloid [94,95]; (3) conversion of PrP^C^ to PrP^Sc^ increases its proteinase K resistance [96], although this feature is not obligatory [97].

To date, data generated by biochemical and biophysical methods, such as spectroscopy analysis, electron microscopy, X-ray fiber diffraction, small-angle X-ray scattering, limited proteolysis, hydrogen/deuterium (H/D) exchange, and surface reactivity measurements, have led to several 3D structural models of PrP^Sc^. However, a recent review exposed discrepancies between the experimental data and these models, indicating that all of these models fail to accommodate the experimental data [98].

### 5.3. Molecular Models for the Structures of PrP^Sc^ and PrP 27–30

The first structural model proposed for PrP^Sc^ was based on Fourier-transform infrared (FTIR) spectroscopy measurements on PrP 27–30. This model predicted a four-stranded β-sheet covered on one face by two *C*-terminal α-helices [99]. However, later experimental observations indicated that PrP^Sc^ appears not to contain any residual α-helical structure [100,101,102].

Downing and Lazo proposed that in PrP^Sc^ β-strands would project from an antiparallel, intertwined β-sheet core, which itself spans the height of eight β-strands equivalent to ~38.4 Å [103]. However, this model contradicts the X-ray fiber diffraction data, which indicated a molecular height of ~19.2 Å [95,98]. Furthermore, this unusual model is different from any known protein fold in the PDB database, which makes it difficult to assess its plausibility.

Based on electron micrographs of 2D crystals of PrP 27–30 and a redacted miniprion, PrP^Sc^106, Govaerts and colleagues proposed a parallel β-helix model for the core of the infectious conformer [104,105]. The authors detected a compatibility between the *N*-terminal region of PrP 27–30 (amino acids 89–176) and a parallel β-helix structure, and proposed that this region of PrP could get converted into a triangular β-helix containing four turns (four-rung model). Furthermore, they proposed that the β-helix associated with an intact *C*-terminal α2-α3 bundle, which retained its α-helical structure from the PrP^C^ fold. Individual PrP 27–30 models were then assembled into a trimeric unit (p3 symmetry) by using the sidewall of the β-helix as an interface. The β-helical PrP trimer can then easily be stacked to form amyloid fibrils. As mentioned above, the *C*-terminal α-helices that are retained in the model are no longer supported by experimental data [100,101,102].

Using molecular dynamics (MD) simulations and sequence comparisons, Langedijk and colleagues [106] suggested the next and alternative model. It proposed a reduced number of β-helix rungs per PrP^Sc^ molecule going from four to two rungs with eight residues in one edge of the triangle, in contrast to the five residues proposed in the previous model [104]. This model helped to overcome problems with the packing density involving a stretch of the PrP sequence (amino acids 106–126) that is rich in small amino acids (glycine and alanine). However, the drawbacks for this model are: First, this model keeps the *C*-terminal α-helices, which are no longer supported by experimental data. Second, the reduction in the number of β-helix rungs results in a molecular height of 9.6 Å for each molecule, which contradicts the experimentally observed molecular height of 19.2 Å (corresponding to four β-helix rungs) [95].

DeMarco and Daggett proposed a “spiral model” where all three α-helices from the original PrP structure were retained, while the number of β-strands was extended to four [107]. Using the structure of recPrP as a starting point, the conformational fluctuations during its conversion under amyloidogenic conditions (acidic pH) were simulated using molecular dynamics. The resulting model proposed a spiraling core of extended structure, consisting of three short β-strands (spanning amino acids 116–119, 129–132 and 160–164) and the recruitment of a nascent β-strand (amino acids 135–140). The molecular dynamics results suggested that the conformational change occurred in the *N*-terminal region of the folded core of PrP, while the *C*-terminal α2 and α3 helices remained intact. In a fibrillar model, which was built up from individual monomers, the β-strands are oriented at angles that are not perpendicular to the fibril axis, which made this model incompatible with the X-ray fiber diffraction results. Similarly, this model contains of high proportion of α-helical structure, which again is inconsistent with recent experimental results [100,101,102].

Tattum and colleagues used electron micrographs of amyloid fibrils grown *in vitro* from recPrP (amino acids 91–231) to generate a 3D reconstruction [108]. The authors found densities of two intertwined protofilaments with a repeating distance of ~60 Å. The repeating unit along each protofilament suggested a distinct, ladder-shaped fibril structure. Other researchers have supported this ladder-shaped model, by proposing that both the *N*-terminal region (amino acids 89–143) and the *C*-terminal region (amino acids 154–227) of the folded core of PrP were prone to conversion into β-structure independently [109,110]. A common structural trait for these models is the presence of a long unstructured loop connecting *N*- and *C*-terminal β-helical domains, which were introduced to fit with the ladder-like appearance of the recPrP amyloid fibers. However, this ladder-like appearance is exclusive to this particular sample preparation, which has not been reported to generate infectivity. Furthermore, the ladder-like configuration has not been found in PrP^Sc^ or PrP 27–30 fibril samples [98].

Cobb and colleagues analyzed recPrP amyloid using site-directed spin labeling and electron paramagnetic resonance (EPR) [111]. Based on interresidue distances (structural constraints), they proposed a parallel in-register β-sheet model, where the recPrP amyloid consists of β-strands and relatively short turns and/or loops with no residual α-helices. Furthermore, each molecule of PrP contributes only with 4.8 Å to the length of the amyloid fibril. The authors also postulate that the *C*-terminal region (amino acids 159–219 in mouse PrP) is converted to a hairpin structure and that the fibrils are formed as a long two-layered β-sheet. Although some researchers have adopted this model to explain the structure of PrP^Sc^ [112], this recPrP amyloid preparation has not been reported to produce infectivity and the model does not correlate with the repeating unit size of 19.2 Å observed by X-ray fiber diffraction experiments on PrP^Sc^ and PrP 27–30 [98].

Similarly, a recent study using solid-state NMR spectroscopy (ssNMR) to analyze the formation of recPrP amyloid fibrils that were seeded with PrP^Sc^, proposed that the *C*-terminal, cysteine-flanked core that contains the α-helices 2 and 3 also adopts a parallel in-register β-sheet architecture [113]. The authors determined the space between labeled isoleucine (Ile-1-^13^C), phenylalanine (Phe-1-^13^C), and leucine (Leu-1-^13^C) residues in recPrP amyloid and observed a distance of ~0.5 nm for four Ile residues (182, 184, 203 and 205). Based on this observation, the authors concluded that these residues refold from a α-helical structure into a parallel in-register intermolecular β-sheet. The three Phe residues (140, 174 and 197) showed an intermolecular distance of 0.5–0.6 nm, suggesting that two‑thirds of the Phe residues were stacked in-register, but that one third was not. However, these findings do not correlate with the molecular height of 19.2 Å that has been observed in PrP^Sc^ and PrP 27–30 amyloid fibrils [95]. Furthermore, it has not been shown to what degree PrP^Sc^-seeded recPrP amyloid resembles brain-derived PrP^Sc^ with respect to infectivity and structure.

## 6. Conclusions

A key question in understanding prion diseases is to elucidate the high-resolution structure of PrP^Sc^. Furthermore, understanding the structural changes that occur during the transition from PrP^C^ to PrP^Sc^ may explain its toxicity and the molecular basis of *in vivo* propagation. Since the initial discovery of prions over 30 years ago, prion research has greatly progressed due to the development of new biochemical and biophysical analysis techniques. Well over thirty mutations have been identified in the *PRNP* gene that are associated with various prion diseases [114,115,116]. Some of these mutations have a pathological effect by directly altering the structure of the protein. However, some of these variations are silent, *i.e.*, without any direct effect on the structure of the protein, but may affect a person’s risk of developing a prion disease. Many of the mutations change a single residue, while others insert additional amino acids or result in a truncated version of the protein. Ultimately, the discovery of these mutations has provided valuable insights on the mechanism of propagation and the conversion of the prion protein.
